# The integrin-adhesome is required to maintain muscle structure, mitochondrial ATP production, and movement forces in *Caenorhabditis elegans*

**DOI:** 10.1096/fj.14-259119

**Published:** 2014-12-09

**Authors:** Timothy Etheridge, Mizanur Rahman, Christopher J. Gaffney, Debra Shaw, Freya Shephard, Jignesh Magudia, Deepak E. Solomon, Thomas Milne, Jerzy Blawzdziewicz, Dumitru Constantin-Teodosiu, Paul L. Greenhaff, Siva A. Vanapalli, Nathaniel J. Szewczyk

**Affiliations:** *Department of Sport and Health Science, College of Life and Environmental Sciences, and ^§^College of Engineering, Mathematics and Physical Science, University of Exeter, Exeter, United Kingdom; Departments of ^†^Chemical Engineering and ^‖^Mechanical Engineering, Texas Tech University, Lubbock, Texas, USA; ^‡^Medical Research Council/Arthritis Research UK Centre for Musculoskeletal Ageing Research, Schools of Life Sciences and Medicine, University of Nottingham, Nottingham, United Kingdom; and ^¶^School of Veterinary Medicine and Science, University of Nottingham, Leicestershire, United Kingdom

**Keywords:** dystrophy, mitochondria, calpain, focal adhesion

## Abstract

The integrin-adhesome network, which contains >150 proteins, is mechano-transducing and located at discreet positions along the cell-cell and cell-extracellular matrix interface. A small subset of the integrin-adhesome is known to maintain normal muscle morphology. However, the importance of the entire adhesome for muscle structure and function is unknown. We used RNA interference to knock down 113 putative *Caenorhabditis elegans* homologs constituting most of the mammalian adhesome and 48 proteins known to localize to attachment sites in *C. elegans* muscle. In both cases, we found >90% of components were required for normal muscle mitochondrial structure and/or proteostasis *vs*. empty vector controls. Approximately half of these, mainly proteins that physically interact with each other, were also required for normal sarcomere and/or adhesome structure. Next we confirmed that the dystrophy observed in adhesome mutants associates with impaired maximal mitochondrial ATP production (*P* < 0.01), as well as reduced probability distribution of muscle movement forces compared with wild-type animals. Our results show that the integrin-adhesome network as a whole is required for maintaining both muscle structure and function and extend the current understanding of the full complexities of the functional adhesome *in vivo*.—Etheridge, T., Rahman, M., Gaffney, C. J., Shaw, D., Shephard, F., Magudia, J., Solomon, D. E., Milne, T., Blawzdziewicz, J., Constantin-Teodosiu, D., Greenhaff, P. L., Vanapalli, S. A., Szewczyk, N. J. The integrin-adhesome is required to maintain muscle structure, mitochondrial ATP production, and movement forces in *Caenorhabditis elegans*.

The integrin-adhesome functions in most cell types to mechanically couple the cytoskeleton with surrounding tissues to provide physical rigidity, transmit contractile forces, and coordinate metabolism to the demands of the external environment in a mechano-sensitive manner (1, 2). This mechano-sensitivity should make integrin-adhesomes particularly important for the regulation of skeletal muscle, a primary function of which is to produce mechanical force for movement. Indeed, clinical consequences of mutations in some integrin-adhesome encoding genes include progressive muscular dystrophy, functional disability, and early death (3–5). Integrin-adhesome components may also regulate normal physiologic muscular adaptations. For example, focal adhesion kinase has been implicated in regulating exercise-induced muscle growth (6, 7) and disuse atrophy (8).

Certain adhesion proteins have previously been reported as essential for embryonic muscle development (9) and sarcomere organization of adult muscle (10, 11) in *Caenorhabditis elegans*. Continuing this, we recently demonstrated that 15 *C. elegans* integrin-adhesome components form a muscle intrinsic maintenance system, the disruption of which compromises the integrity of muscle adhesomes, sarcomeres, mitochondria, and cytosolic proteostasis (12). However, this muscle maintenance system is likely much more elaborate. Structural examination indicates adhesomes are electron-dense in *C. elegans* (13) and human (14) muscle, signifying the congregation of large amounts of proteins at these sites. Congruent with this, a consolidation of histologic and functional genomic integrin-adhesion research from various mammalian cell types lists 151 proteins as part of integrin-adhesion complexes (15). These include key structural proteins, Rho family GTPases, protein kinases, lipid kinases, phosphatases, and proteases, all of which are involved in nearly 700 protein-protein interactions (15). Integrin-adhesomes therefore possess the capacity to regulate muscle not only *via* provision of structural integrity but also by orchestrating a complex interplay between cytoskeletal structure, metabolism, and signaling pathways. Whether the pattern of muscle subcellular defects observed in our previous disruption of 15 adhesion genes (12) extends across disruption of the entire adhesome is, however, unknown. Additionally, whether these morphologic abnormalities translate to functional impairment of muscle metabolic capacity and movement force production remains to be determined. Such functional characterization of the adhesome will advance understanding of both the normal maintenance of muscle homeostasis and the pathogenesis of muscular dystrophies.

Systematic evaluation of the 151 adhesome components and their role in maintaining muscle health within mammalian systems would take many years at significant expense. Thus, using alternative model systems is appropriate for expediting understanding of the importance of adhesomes in muscle. The invertebrate *C. elegans* is a genetically tractable organism that allows rapid *in vivo* exploration of signaling systems using forward and reverse genetics, combined with concomitant analysis of their physiologic/functional relevance. Importantly, sarcomere architecture is almost identical between *C. elegans* and mammals (16) and many of the major molecular signaling pathways (17) and muscle protein degradation pathways (18) are present in both systems. *Caenorhabditis elegans* are, therefore, highly suited to the rapid exploration of novel biological pathways. Thus, this study used *C. elegans* to knock down, using RNA interference (RNAi), *C. elegans* putative homologs of each of the 151 proteins comprising the mammalian integrin-adhesome network and analyze the effects on muscle subcompartment structure. We further used representative adhesome mutants to assess the consequences of adhesome failure on mitochondrial function and movement force production.

## MATERIALS AND METHODS

### RNAi screening protocol

Nematode strains were maintained and grown at 20°C as previously described (19) using the *Escherichia coli* strain OP50 as food source. Transgenic strains were used to report on cytosolic protein content [*ccIs55*: *sup-7*(*st5*); *unc-54::lacZ*; integrated on linkage group (LG) V] with visualization by histology as previously described (20), and green fluorescent protein (GFP)-tagged mitochondria [*ccIs4251*: pSAK4 (myo*-3* promoter driving mitochondrially targeted GFP); pSAK2 (myo*-3* promoter driving a nuclear-targeted GFP::LacZ fusion); and a *dpy-20* subclone; integrated on LG I], sarcomeres [*jIs01*: *rol-6*(*su1006*); myo*-3::gfp*; unknown site of integration], and adhesome [*ryIs22*: *rol-6*(*su1006*); *unc-95::gfp*; integrated on LG X] with visualization by epifluorescence microscopy as previously described (12).

RNAi treatments using bacterial feeding vectors were performed essentially as previously described (21). Most bacterial lawns expressing double-stranded RNA were grown from bacterial clones from the MRC Ahringer Library or from the Open Biosystems Vidal Library, with the exception of *pat-3* (12). All RNAi clones were checked for sequence verification against the intended target gene. All RNAi experiments were conducted alongside empty vector controls for all strains tested. Following the expected (12) high number of observed muscle defects with RNAi knockdown of adhesome genes, we conducted additional negative and positive control RNAi gene knockdowns. For negative controls, RNAi was conducted against a series of 10 non–adhesome-related genes whose knockdown is known to confer a behavioral phenotype (*i.e.,* uncoordinated movement of animals) but no submuscular abnormalities (18). The genes targeted for negative controls, plus the sequence-verified clone used to conduct RNAi, are as follows: *unc-10* (X-3F06); *unc-11* (I-1J11); *unc-13* (I-3N19); *unc-14* (I-3B19); *unc-29* (I-4M14); *unc-30* (IV-7A23); *unc-38* (I-2M15); *unc-55* (I-3L14); *unc-101* (I-6G20); and *unc-105* (II-6A05). For potential positive controls, acute RNAi knockdown was conducted in adults targeting 10 non-adhesome genes whose knockdown produces an embryonic lethal phenotype in response to knockdown during development. The genes targeted for positive controls, plus the sequence-verified clone used to conduct RNAi, are as follows: *icd-1* (10001 B6), *snr-6* (10001 E1), *npp-20* (10001 E5), *vha-9* (10001 G2), *nap-1* (10002 B6), *ufd-1* (10002 C9), *fnta-1* (10002 F7), *pbs-2* (10002 G3), *cyb-2.1* (10002 H7), and *crn-1* (10003 A3).

#### RNAi against C. elegans homologs of the mammalian integrin-adhesome

RNAi clones used for each *C. elegans* homolog of mammalian adhesome components can be viewed in Supplemental Fig. S1. Bioinformatic work was conducted using Ensemble (22), Wormbase (23), and Ortholist (24). For mammalian adhesome components with multiple putative *C. elegans* homologs, all homologs were knocked down by RNAi (total number of genes = 166; additional data not presented). Because subsequent analysis in Cytoscape software is dependent on input of a single putative homolog, the result with the most severe RNAi-induced phenotype (if any) was taken as the single homolog for inclusion in the final analysis presented herein. The expression of integrin-adhesome components specifically within muscle was determined by searching each component in Human Protein Atlas (25).

#### RNAi against muscle-specific C. elegans adhesome genes

To assess the consequences of RNAi knockdown of components specifically associated with *C. elegans* muscle, RNAi was also performed against a list of 47 adhesome proteins previously identified as localizing to muscle adhesion complexes in *C. elegans* (26).

**Figure 1.**
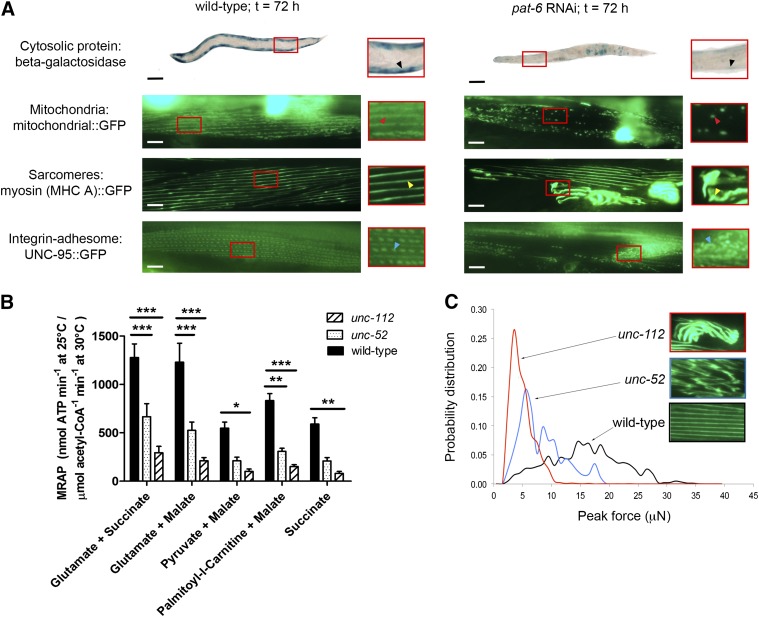
Muscle subcellular dystrophy relates to functional declines in mitochondrial maximal ATP production and voluntary muscle force. *A*) Examples of submuscular phenotypes scored for cytosolic protein content (first row), mitochondria (second row), sarcomeres (third row), and adhesomes (fourth row). The large, circular, overexposed regions in the mitochondrial images are GFP-labeled nuclei and are normal for the strain. Black arrowheads, normal cytosolic protein content (wild-type) and clearly reduced cytosolic report protein (*pat-6* RNAi); red arrowheads, normal mitochondrial reticulum (wild-type) and near complete loss of reticular mitochondria (*pat-6* RNAi); yellow arrowheads, normal myosin filament striation (wild-type) and myosin disorganization (*pat-6* RNAi); blue arrowheads, normal integrin adhesion sites (wild-type) and loss of adhesion complex organization (*pat-6* RNAi). Black bars, 100 *μ*m; white bars, 10 *μ*m. *B*) MRAP for wild-type animals *vs*. *unc-52* and *unc-112* mutants (*n* ∼ 250 animals per condition). Values represent means ± sem. Using 2-way mixed-model ANOVA with Bonferroni *post hoc* tests for pairwise comparisons: *P < 0.05; **P < 0.01; ***P < 0.001. *C*) Voluntary muscle force production and representative sarcomere structure in wild-type animals *vs*. *unc-52* and *unc-112* mutants.

To assess the effects of RNAi gene knockdown in fully differentiated adult muscle, animals were first roughly age-synchronized at the L1 larval stage as previously described (20). Animals were then replated to fresh OP50 bacterial lawns and grown at 16°C for 48–60 h to young adulthood. After confirmation of normal behavioral and submuscular phenotypes in all reporter strains at young adulthood (*t* = 0 h), animals were manually transferred to RNAi plates seeded with standard *Escherichia coli* HT115 RNAi feeding vectors, as described above. Animals were then maintained at 20°C for 72 h until midlate adulthood, ensuring animals remained well fed by transferring to fresh RNAi plates between 24 and 48 h after young adulthood. Analysis of behavioral and submuscular phenotypes was performed at 24, 48, and 72 h after young adulthood. At each time point, 20 animals were analyzed for each transgenic report strain: cytosolic protein content by histology and mitochondrial localization, sarcomere structure, and adhesome localization by epifluoresence microscopy (using a Zeiss AX10 with an Axiocam MRC digital camera and Axiovision LE software; Carl Zeiss, Göttingen, Germany). The additional positive and negative control RNAi experiments were analyzed using a Nikon H600L microscope (Nikon Instruments, Tokyo, Japan). Examples of the muscle phenotypes scored are provided in **Fig. 1*A***.

To determine the effects of RNAi gene knockdown during animal development, RNAi treatments were conducted by placing 3–4 L4-stage larvae hermaphrodite worms (P_0_) from each transgenic reporter strain onto separate nematode growth medium RNAi plates (made in the laboratory in accordance with ref. 19) seeded with bacteria lawns expressing double-stranded RNA for the relevant genes and then incubating for 72–96 h at 20°C when the first generation progeny of P_0_ animals had reached young adulthood. The first generation was analyzed for behavioral and submuscular phenotypes as described above at young adulthood (*t* = 0 h) and 24, 48, and 72 h. L4 larvae hermaphrodites from the first generation were then transferred to newly seeded nematode growth medium RNAi plates with the same bacteria for examination of the second generation as above. The behavioral phenotypes observed are provided in Supplemental Fig. S1.

### Scoring of behavioral and submuscular phenotypes

The behavioral phenotypes scored during RNAi treatments were Bmd (abnormal body morphology), Clr (translucent body), Dpy (short fat appearance), Egl (egg laying defective), Emb (embryonic lethal), Gro (long period of development and/or growth arrest), Let (lethal), Lva (larval arrest), Lvl (larval lethal), Prl (paralyzed), Pvl (protrusion from the vulva), Reduced brood size, Slu (sluggish movement), Ste (sterile), and Unc (uncoordinated movement) (see OBO Foundry website for complete definition of all *C. elegans* behavioral phenotypes) (27). Owing to the inherent variability of RNAi feeding vector experiments, the reliability and reproducibility of our RNAi results was confirmed by comparing the observed behavioral phenotypes to previously published reports for the same RNAi clone, described in detail in ref. 18. This analysis revealed that 84% of the behavioral phenotypes observed in the present RNAi screen were consistent with past studies, whereas 7% of our observations appear to be the first report of a behavioral phenotype for knockdown of the targeted gene and 10% of our observations do not confirm past reports. These values are similar to other large-scale RNAi screens from our laboratory (18, 28) and suggest that the level of variability in RNAi knockdown is roughly 10%, on par with many standard techniques such as Western blotting (29).

The submuscular phenotypes for cytosolic protein content, mitochondrial localization, sarcomere structure, and adhesome localization were scored as either normal or abnormal. Cytosolic protein content was scored as abnormal if a loss of *β*-galactosidase staining was observed *vs*. wild-type controls (12). Mitochondrial defects were scored as widespread gaps and/or fragmentation of mitochondrial::GFP. Sarcomere abnormalities were scored as disorganized, fraying, and/or balling of myosin heavy chain A::GFP. Adhesome defects were scored as gaps, disorganization, or fraying/balling of uncoordinated (UNC)-95::GFP (12). Defects in ≥2 muscle cells had to be observed for scoring as abnormal in each GFP reporter protein screen. Fig. 1*A* shows normal and abnormal examples of each muscle compartment. For muscle phenotypes to be scored as abnormal at individual time points, defects had to be observed in ≥50% (for cytosolic protein, mitochondria, and sarcomeres) or 30% (for adhesomes) of worms analyzed. These thresholds were set higher than previous RNAi screens (*i.e.,* 20–25% defect acceptance threshold) (18, 30) and in line with others (31) to reduce the likelihood of overscoring muscle abnormalities as might be anticipated from previous reports of adhesome gene knockdowns (12). The lower threshold for adhesome defects reflects that, in our hands, this reporter is more resistant to muscle abnormalities in positive control RNAi knockdowns (*unc-112* and *pat-4;* clones used as in ref. 12). For acute RNAi treatments in adult animals, for phenotypes to be classified as abnormal for the final analysis, abnormal muscles had to be observed for ≥2 time points and/or 72 h only. For developmental RNAi experiments, abnormal phenotypes had to be observed for ≥2 time points in both first- and second-generation animals or 4 time points in a single generation. Observation of abnormal muscles only at the 72 h time point was not scored as abnormal for that RNAi clone for developmental RNAi treatments. Additionally, muscle defects were scored for the final analysis if the above criteria was met for both chronic and acute experiments; developmental RNAi treatments only; or in acute RNAi treatments only if muscle defects were observed at at least the 48 and 72 h time points (*i.e.,* abnormal phenotypes were not scored if only observed at 72 h acutely and not developmentally). A summary of behavioral and submuscular phenotypes for all *C. elegans* homologs of the mammalian adhesome targeted by RNAi can be viewed in Supplemental Fig. S1.

### Maximal rates of mitochondrial ATP production

To determine the effects of integrin-adhesome dysfunction on maximal rates of mitochondrial ATP production (MRAP), representative temperature-sensitive adhesome mutants *unc-52* [*su250*^ts^ II; *ccIs55*(*unc-54::lacZ*) V] and *unc-112* [*r367*^ts^; *ccIs55*(*unc-54::lacZ*) V] were used alongside wild-type *ccIs55* controls. Animals were roughly age synchronized as previously described (20) and then replated to fresh OP50 bacterial lawns and grown at 16°C for 48–60 h to early adulthood. Subsequently, animals were transferred to 25°C and analyzed at 72 h after adulthood.

The measurement of MRAP was based on the method for measuring MRAP in isolated mitochondria (32). Animals were washed from plates using M9 buffer and were washed using centrifugation. The final wash was completed with homogenization buffer (100 mM KCl, 50 mM KH_2_PO_4_, 50 mM Tris, 5 mM MgCl_2_, 1 mM EDTA, and 1.8 mM ATP at pH 7.2) to dilute the presence of M9. Animals were resuspended in 300 μl homogenization buffer containing saponin at 1 mg/ml and were homogenized with a teflon pestle (Kontes glass) for 3 min at 200 rpm. The homogenate was centrifuged at 650 *g* for 3 min to remove intact worms and nuclear fractions. The supernatant was removed and then centrifuged at 15,000 *g* for 3 min to pellet the mitochondria. The supernatant was removed and discarded, and then the pellet was resuspended in 300 *μ*l homogenization buffer without ATP (100 mM KCl, 50 mM KH_2_PO_4_, 50 mM Tris, 5 mM MgCl_2_, and 1 mM EDTA at pH 7.2). The mitochondrial suspension was further centrifuged at 15,000 *g* for 3 min to wash the mitochondria. The mitochondrial pellet reformed, and after removing the supernatant, the pellet was added to 300 *μ*l of resuspension buffer [human serum albumin, 0.5 mg/ml, 240 mM sucrose, 15 mM KH_2_PO_4_, 2 mM Mg(CH_3_COO)_2_ × 4 H_2_O, and 0.5 mM EDTA at pH 7.2] and was centrifuged at 15,000 *g* for 3 min as a further wash stage for the mitochondria. The final mitochondrial pellet was added to 100 *μ*l of resuspension buffer and remained on ice until analyzed.

MRAP was determined luminometrically as previously described (32). Mitochondria were diluted (1:400) before 2.5 *μ*l of diluted mitochondrial suspension was added to each well. MRAP were calculated from the change in luminescence delivered by 150 pM ATP standard. Citrate synthase (CS) activity was determined to normalize MRAP for variation in mitochondrial content (32, 33). To measure CS activity, 15 *μ*l mitochondrial suspension was added to 185 *μ*l homogenization buffer (50 mM KH_2_PO4, 1 mM EDTA, and 0.05% Triton X-100) and was homogenized using a glass pestle at 200 rpm for 2 min. The homogenate was then centrifuged at 24,000 *g* before the formation of 5,5′-dithiobis-(2-nitrobenzoic acid)-coenzyme A containing a thiol group (DTNB-CoA-SH) in the supernatant was measured spectrophotometrically at 412 nm to determine CS activity (32, 33).

### Quantification of *C. elegans* movement forces

To assess muscle movement forces, we characterized gross muscle force production in wild-type animals and *unc-52* and *unc-112* mutants (same alleles as for MRAP experiments described above). The movement force generation assay is based on deflection of soft microfabricated pillars by moving worms (34, 35) as shown in Supplemental Fig. S2. Worms (young adult) were pipetted into the force-measurement device, and images were recorded at 28 frames/s for 0.5 min for each worm. The displacement of the pillars in an image was measured using a custom curve fitting code written in Matlab (R2013b; Mathworks, Natick, MA, USA). The individual pillar displacements were converted into the corresponding forces using a modified form of the Timoshenko beam deflection theory (36). To generate the probability distribution function of forces shown in Fig. 1*C*, we extracted the peak (or maximal) force (*f_p_*) exerted by the animal in each image and binned the *f_p_* data acquired from all images and all animals. At least 15 animals per strain and >6800 *f_p_* data points were used to generate a probability distribution function. All experiments were conducted in synchronous young adult animals at a temperature of 20 ± 1°C to avoid the paralysis that occurs in *unc-52* and *unc-112* mutants with age and also following shifting to the nonpermissive 25°C (both strains display uncoordinated movement and sarcomere defects at young adulthood at the permissive 20°C).

### Analysis

Cytoscape 2.8.3 software was used to cluster and present phenotype results and display interactions between individual adhesome components. The keys used for this Cytoscape analysis are as follows. Interactions: green connections show activation of downstream interacting component; red connections show inhibition of downstream interacting component; and black connections show physical binding between components. Component shape: rectangle shows adaptor proteins; parallelogram shows G protein–related proteins (GTPase, GAP, GEF); diamond shows Tyr or Ser/Thr kinases; V-shape shows transmembrane proteins; triangle shows actin/actin regulators; hexagon shows Tyr or Ser/Thr phosphatases; circle shows lipids/lipid metabolism proteins; octagon shows chaperones or protein degradation elements; and rounded rectangle shows DNA/RNA regulators. Border color: black borders show intrinsic components and white borders show associated components. Component fill color: red shows structural defect; blue shows metabolic defect; and white shows no phenotype. MRAP data were analyzed using a 2-way mixed-model ANOVA with Bonferroni *post hoc* tests for pairwise comparisons. Statistics were completed using GraphPad Prism (GraphPad, La Jolla, CA, USA).

## RESULTS

### Most mammalian integrin-adhesome components have putative homologs in *C. elegans*

Ensemble (22) and Wormbase (23) searches identified 123 putative *C. elegans* gene homologs of the 151 mammalian integrin-adhesome proteins (15), signifying 81.5% conservation between species. Sequence verified, commercially available RNAi clones were available against 113 of the 123 identified homologs, and 90% of these putative homologs match homology were assigned by a meta-review (Ortholist) (24). The 151 mammalian adhesome components have been further categorized, using adhesion-localization data, into 90 core constituents that physically reside within integrin-adhesomes and 61 associated components that transiently interact with the core components (15). Using these categories, 66 *C. elegans* gene homologs are classified as core, components and 47 are associated adhesome components (categorization of specific adhesome components are included in Supplemental Fig. S1).

### Most integrin-adhesome proteins are required for muscle structural and metabolic integrity

**Figure 2.**
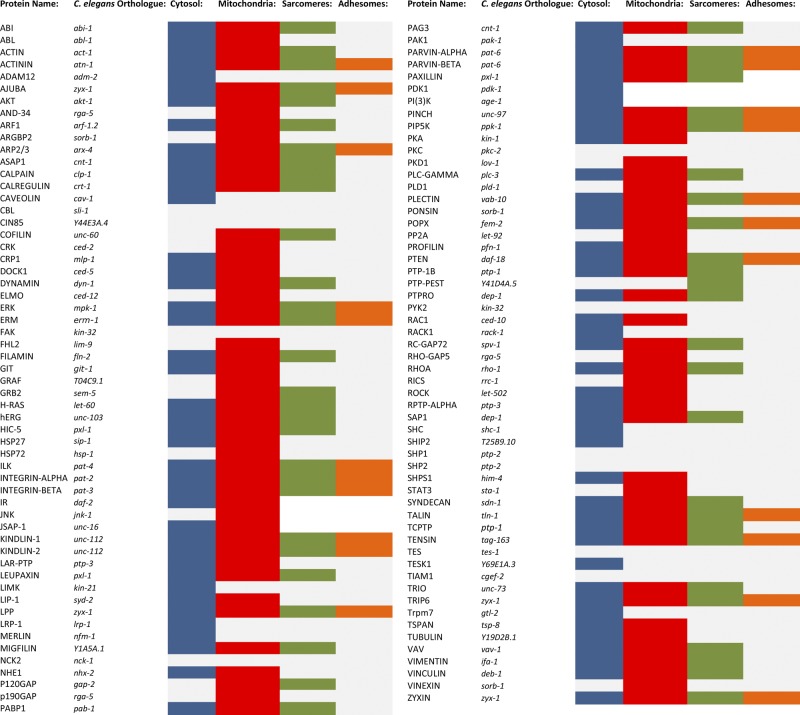
Submuscular phenotypes observed with RNAi knockdown of each of the 113 *C. elegans* homologs of the mammalian adhesome. Colors represent RNAi-induced muscle subcompartment phenotypes: blue, loss of cytosolic protein content; red, mitochondrial fragmentation; green, sarcomere disorganization; orange, abnormal adhesome structure; gray, no phenotype.

**Figure 3.**
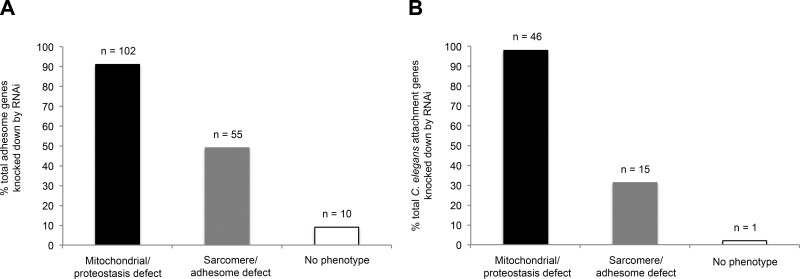
Proteins known to localize to *C. elegans* muscle adhesion sites are required for maintained muscle integrity. *A*) Number of mitochondrial/proteostasis and sarcomere/adhesome defects observed after RNAi knockdown of *C. elegans* homologs of the mammalian adhesome. *B*) Number of mitochondrial/proteostasis and sarcomere/adhesome defects observed after RNAi knockdown of components that localize to adhesion sites in *C. elegans*.

Representative images of the phenotypes scored herein for each submuscular compartment can be viewed in Fig. 1*A*, and comparative sarcomere defects in loss of function adhesome mutants can be viewed in Fig. 1*C*. A summary of results for each of the 113 individual *C. elegans* gene knockdowns is provided in **Fig. 2** (full details are provided in Supplemental Fig. S1). Of the 113 putative *C. elegans* homologs targeted by RNAi, knockdown, 102 homologs (90% of all integrin-adhesome knockdowns) induce a defect in mitochondrial structure and/or in proteostasis. Of these 102 knockdowns, 54 also display disrupted integrin-adhesome and/or sarcomere structure (**Fig. 3*A*** and **Table 1**); only 1 knockdown (PTP-PEST) caused a sarcomere defect without a corresponding mitochondrial and/or proteostasis abnormality, giving a total of 55 knockdowns that induced a sarcomere/adhesome defect. Comparing these 55 genes to 118 genes whose knockdown by RNAi caused myosin::GFP disorganization in a previous RNAi screen of all 3577 genes expressed in *C. elegans* muscle (31) found that 8 knockdowns (15%) were congruent between datasets. Further comparison with previous targeted RNAi studies (12, 18) found 19 gene knockdowns (35%) were in agreement with the present study. Last, extending this comparison to the wider published literature found that, of the 55 gene knockdowns identified to cause sarcomere defects, 42 of these genes (76%) have a known role in regulating actomyosin function in *C. elegans* (37–39).

**TABLE 1.** Individual adhesome components comprising Fig. 3A, B.

Integrin adhesome component			Dense body component		
Mitochondrial/proteostasis defect	Sarcomere/adhesome defect	No phenotype	Mitochondrial/proteostasis defect	Sarcomere/adhesome defect	No phenotype
ABL	ABI	CBL	*D1007.4*	*eva-1*	*W09C9.2*
ADAM12	ACTIN	CIN85	*F25H2.4*	*C47B2.2*	
AND-34	ACTININ	FAK	*F26A3.4*	*F25H2.12*	
ARGBP2	AJUBA	NCK2	*set-18*	*mom-4*	
CAVEOLIN	AKT	PKC	*pfd-2*	*T01G9.2*	
CRK	ARF1	PYK2	*ltd-1*	*hgo-1*	
CRP1	ARP2/3	SHP1	*smg-8*	*ttll-12*	
DOCK1	ASAP1	SHP2	*F42H10.3*	*F22B5.10*	
ELMO	CALPAIN	TES	*cutc-1*	*Y71H2AM.15*	
FHL1	CALREGULIN	TIAM1	*har-1*	*pyk-2*	
GIT	COFILIN		*ZK637.2*	*bre-1*	
GRAF	DYNAMIN		*K08E3.5*	*F22F7.7*	
HSP27	ERK		*aldo-1*	*pkn-1*	
HSP72	ERM		*R102.5*	*W05G11.6*	
IR	FILAMIN		*D2063.1*	*C04G6.4*	
JNK	GRB2		*Y43F8B.2*		
JSAP-1	H-RAS		*tag-303*		
LAR-PTP	hERG		*gei-15*		
LIMK	HIC-5		*C17G1.7*		
LIP-1	ILK		*gyg-1*		
LRP-1	INTEGRIN-*α*		*M79.2*		
MERLIN	INTEGRIN-*β*		*M02D8.1*		
NHE1	KINDLIN-1		*Y39A1C.1*		
p190GAP	KINDLIN-2		*W03F9.1*		
PAK1	LEUPAXIN		*C40H1.6*		
PDK1	LPP		*Rsbp-1*		
PI(3)K	MIGFILIN		*Arrd-15*		
PKA	P120GAP		*Hip-1*		
PKD1	PABP1		*Y57G11C.3*		
PLD1	PAG3		*Y48G10A.3*		
PONSIN	PARVIN-*α*		*Y48G10A.3*		
PP2A	PARVIN-*β*				
PROFILIN	PAXILLIN				
RAC1	PINCH				
RACK1	PIP5K				
RHO-GAP5	PLC-*γ*				
RICS	PLECTIN				
ROCK	POPX				
RPTP-*α*	PTEN				
SHC	PTP-1B				
SHIP2	PTP-PEST				
SHPS1	PTPRO				
STAT3	RC-GAP72				
TESK1	RHOA				
Trpm7	SAP1				
TSPAN	SYNDECAN				
TUBULIN	TALIN				
VINEXIN	TCPTP				
	TENSIN				
	TRIO				
	TRIP6				
	VAV				
	VIMENTIN				
	VINCULIN				
	ZYXIN				

RNAi knockdowns that gave mitochondrial/proteostasis abnormalities include those components listed under both mitochondrial/proteostasis defects and sarcomere/adhesome defects columns (with the exception of PTP-PEST, which induced sarcomere/adhesome defects only).

Because the gene list targeted herein represents a bioinformatically predicted *C. elegans* version of the ubiquitous integrin-adhesome and because the precise molecular composition of this adhesome differs between cell types (15), knockdown-induced muscle defects could be a non–cell-autonomous effect from loss of non-muscle cell adhesion. We therefore examined the 113 adhesome genes knocked down by RNAi against for known expression in *C. elegans* embryonic muscle cells (31) and found that 71 (63%) are expressed in muscle, as identified by both serial analysis of gene expression (SAGE) and microarray analysis or 95 (84%) as identified by SAGE alone. However, the number of adhesome genes expressed in the neurons and gut is 104 (92%), when analyzed by SAGE alone. Human Protein Atlas (25) searches of each mammalian integrin-adhesome component also found that ≥103 are present in muscle. Thus, there is significant scope for the negative effects of adhesome knockdown to be a consequence of impaired adhesion directly in muscle even though non–cell-autonomous effects are also likely. To further test our findings on the bioinformatically predicted integrin-adhesome for muscle homeostasis, we knocked down an additional 47 genes whose protein products are known to localize to *C. elegans* muscle attachment sites (26). The pattern of muscle subcellular defects observed is similar to that found with disruption of the predicted integrin-adhesome: knockdown of 46 genes, 98%, caused a defect in mitochondrial structure and/or proteostasis, 15 of which also caused sarcomere dystrophy (Fig. 3*B* and Table 1). This finding further suggests the large scope for cell-autonomous effects of integrin-adhesome knockdown in muscle.

Given the very high percentage of both integrin-adhesome and muscle attachment gene knockdowns that yielded defects in muscle, we performed RNAi against a set of 10 knockdowns previously shown to produce a movement defect but not a subcellular defect in muscle (subset of genes from ref. 18) and, as expected, failed to find defects in muscle despite finding impaired movement. Next, we performed RNAi against a set of 10 knockdowns previously shown to produce embryonic lethality [*icd-1* (inhibitor of cell death), *snr-6* (small nuclear ribonucleoprotein), *npp-20* (nuclear pore complex protein), *vha-9* (vaculolar ATPase), *nap-1* (nucleosome assembly protein), *ufd-1* (ubiquitin fusion degradation), *fnta-1* (farnesyltransferase), *pbs-2* (proteasome *β*-subunit), *cyb-2.1* (cyclin B), and *crn-1* (cell death related nuclease)]. In each case, we found embryonic lethality as expected. When these genes were knocked down in fully developed adults, we further found both increased protein degradation and loss of mitochondrial reticular structure but no defects in the sarcomere structure. These results suggest the sarcomere defects observed in response to knockdown of integrin-adhesome and muscle attachment genes are not simply a nonspecific effect of disruption of essential cellular functions; the observed increased protein degradation and fragmented mitochondrial reticulum may be general consequences of disrupted essential cellular function.

### Proteins for which knockdown induces structural defects are enriched for core adhesome components that physically interact

**Figure 4.**
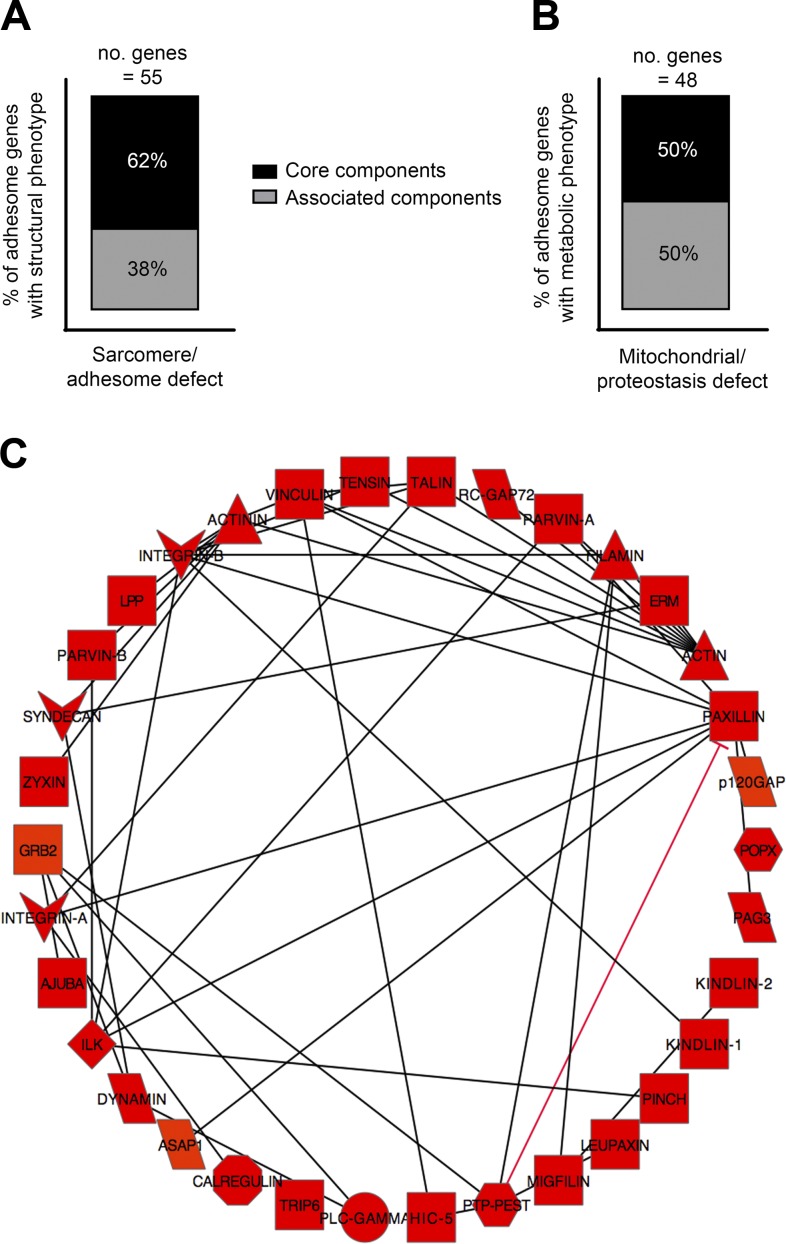
Knockdown-induced sarcomere/adhesome muscle defects are enriched for core adhesome components that physically interact. *A*) Percentage of total sarcomere/adhesome defects induced by RNAi against core and associated adhesome components. *B*) Percentage of total mitochondrial/proteostasis defects induced by RNAi against core and associated adhesome components. *C*) Thirty-one of the 34 core adhesome knockdowns that cause sarcomere/adhesome defects are predicted to physically bind with other core adhesome components. This figure was generated using Cytoscape software.

We have previously shown core proteins to be important for preventing the most severe structural dystrophic phenotypes in muscle (12). We therefore determined whether this trend extends to the complete adhesome. Of the 55 knockdowns that induce sarcomere defects, 34 (62%) are against core elements and 21 (38%) are against associated components, showing enrichment for knockdown of core proteins as causing the most severe muscle defects (**Fig. 4*A***). However, the 48 knockdowns that affect only mitochondrial structure and/or proteostasis are evenly divided between 24 intrinsic (50%) and 24 associated (50%) adhesome components (Fig. 4*B*). Further examination of the type of protein-protein interaction between individual components (as identified in mammalian muscle) (15) reveals that of the 34 intrinsic component knockdowns that give a sarcomere defect, 31 physically interact with ≥1 other intrinsic adhesome component (Fig. 4*C*).

### Knockdown of the calpain protease functional subgroup is enriched for inducing structural defects

**Figure 5.**
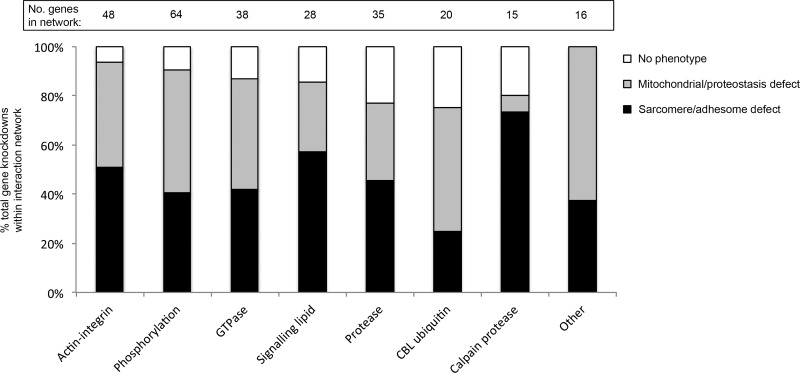
The calpain interaction network is enriched for knockdown-induced sarcomere/adhesome muscle defects. RNAi knockdown of individual adhesome components were classified into interaction networks based on broad functional classes (15) and analyzed for the percent structural, metabolic, and normal phenotypes observed for RNAi knockdown of individual genes contained within each network. Numbers above bars indicate number of genes knocked down within each interaction network.

When the adhesome network subnets of functional classes of interacting proteins (15) are examined, knockdown of constituent components of each subnet resulted in a similar percentage of submuscular defects between the functional classes. Knockdown of the calpain system, however, showed strong enrichment (73%) for sarcomere and/or adhesome defects (**Fig. 5**). This is higher than the 52% of core adhesome components whose knockdown caused sarcomere/adhesome disruption or the 40 ± 14% (mean ± sd) sarcomere/adhesome defects found with knockdown of individual components comprising the largest interaction networks (classed here as adhesome components with 10–45 interacting partners, a total of 44 of 113 components).

### Adhesome dysfunction impairs mitochondrial maximal ATP production and generation of movement force

MRAP and movement forces were measured in animals possessing either of 2 integrin-adhesome component mutations: *unc-52*/perlecan or *unc-112*/kindlin. These mutants were selected because they represent collapse at the level of the extracellular matrix interface (*unc-52*) and intracellular interface (*unc-112*). Both mutants display reduced rates of maximal mitochondrial ATP production with glutamate + succinate as a substrate (667 ± 428, 294 ± 212, and 1278 ± 634 nmol ATP min^−1^ at 25°C /*μ*mol acetyl-CoA^−1^ min^−1^ at 30°C at 30°C for *unc-52, unc-112*, and wild-type animals, respectively; *P* < 0.001; Fig. 1*B*). Maximal ATP production was also significantly reduced in both mutants when glutamate + malate and palmitoyl-*l*-carnitine + malate were used as mitochondrial substrates (*P* < 0.05). Only *unc-112* mutants displayed lowered maximal ATP production when pyruvate + malate and succinate were used as substrates (*P* < 0.05). Development of movement force was also decreased in both *unc-52* and *unc-112* adhesome mutants, as indicated by a reduced probability distribution of muscle movement forces *vs*. wild-type animals (Fig. 1*C*).

## DISCUSSION

The importance of some integrin-adhesome proteins in maintaining both muscle structural integrity and intracellular signaling has recently gained increased appreciation. However, the full mammalian integrin-adhesome network comprises ≥151 proteins, the requirement of which for maintaining muscle subcellular structure, mitochondrial capacity, and movement strength is unknown. Here we show for the first time that the majority of adhesome components are required for maintained muscle metabolic homeostasis and that approximately half of these are also necessary to preserve sarcomere structure. We also demonstrate that loss of adhesome integrity translates to impaired muscle function at the levels of mitochondrial ATP production and movement force capacity.

### Muscle maintenance by the complete integrin-adhesome

Because adhesome proteins physically tether mitochondria (40) and regulate protein synthetic pathways (41), it is perhaps not surprising that knockdown of adhesome genes results in abnormal mitochondrial structure and perturbed proteostasis. Indeed, a restricted number of mammalian adhesion proteins have been shown to regulate metabolic function, including insulin-mediated signaling (41, 42), protein synthesis (6, 7), protein degradation (12), and mitochondrial localization (12, 43). The present results, however, demonstrate that the vast majority of individual components of the integrin-adhesome are each required for maintained mitochondrial structure and proteostasis. Although we have not demonstrated that it is the knockdown of each of the integrin-adhesome components within muscle that induces muscle defects, 2 lines of evidence suggest that it is likely that the knockdowns are acting within muscle. First, a large number of the integrin-adhesome components are known to be expressed in both *C. elegans* muscle and human muscle. Second, we observed a similar pattern of submuscular defects when knocking down known *C. elegans* muscle attachment genes (26). Although this does not refute the possibility that nonmuscle RNAi effects contribute to muscular decline, a high number of adhesome genes are also expressed in neuronal and gut tissue, and there is clear capacity for the reported dystrophic phenotypes to be caused by loss of intrinsic muscle adhesion. Regardless of cell autonomy, the large extent to which each of the components of the integrin-adhesome is required for muscle maintenance suggests that integrin complexes have an important metabolic role and that little redundancy exists within the integrin-adhesome gene network. The importance of each component of the integrin-adhesome for muscle maintenance contrasts with other large-scale RNAi knockdown screens, for example, of the *C. elegans* kinome (18, 30), again suggesting little functional redundancy in the integrin-adhesome. However, the importance of each component seems similar to our limited knockdown of essential genes, which is consistent with adhesomes being an essential cellular structure. Additionally, our results indicate adhesome complexes as a whole, including numerous cell signaling molecules, are more important for maintained sarcomere structure than suggested by the limited number of individual components identified as mutated in patients with muscular dystrophies, such as the heterodimeric *αβ* integrins (3) and calpain 3 (4). The high number of adhesome genes whose knockdown cause sarcomeric defects may, in fact, be an underestimation. For example, here we assessed myosin structure, but certain adhesome mutations can preferentially disturb actin organization (44, 45). Additionally, the reporter used for monitoring adhesome structure (UNC-95::GFP) is not a conserved protein and is an associated component of the adhesome (15). It is therefore possible that UNC-95 as a reporter underestimated the structural role of other conserved adhesome proteins; indeed, RNAi against the downstream UNC-95 effector, paxillin (PXL-1) (46), results in normal adhesome structure. Regardless, the regulation of muscle structural and metabolic integrity by integrin-adhesomes appears to be much more intricate and extensive than previously suggested by studies of the roles of individual adhesion components.

### Role of adhesome functional subgroups in maintaining muscle integrity

Previous studies have shown core adhesome proteins to be most important for preventing severe structural dystrophic phenotypes in muscle (12). Here we report that this pattern extends across the entire adhesome, with knockdowns that give both metabolic and structural defects being enriched for core proteins, nearly all of which physically interact with ≥1 other core adhesome component. These findings suggest that loss of physical binding between core components may cause a central collapse of the adhesome 3-dimensional structure. The impact of such a collapse would therefore have catastrophic consequences for muscle integrity as the result of failure of not a single, linear pathway but rather the combined failure of the numerous interconnected networks present in the adhesome. One prediction of central collapse of the integrin-adhesome is that repair and/or replacement of failing attachments should take place. Thus, it is perhaps unsurprising that of the functional subclasses present within the adhesome (15), only knockdown of the calpain proteolytic interaction network that acts to repair and/or replace attachments (47) was enriched for causing severe structural dystrophy. Consistent with the nature of specific interactions being key to the relative importance of adhesome components for maintaining muscle integrity, adhesome components with the highest number of interacting partners are not more likely to induce the more severe structural muscle abnormalities. Thus, core physically interacting proteins and the calpain subgroup of the adhesome appear to display less functional redundancy than in other subnets or in the largest interaction networks. This is congruent with, and extends the scope of, previous reports (48–52) that calpains and their associated signaling pathways appear to play a central role in the regenerative capacity of muscle as a consequence of the need to maintain integrin-adhesome complexes.

### Effects of dysfunctional adhesomes on muscle function

Movement defects (12, 53, 54) and reduced maximum bending amplitude, reflective of impaired force transmission (44, 55), have previously been reported in *C. elegans* in response to integrin-adhesome disruption. However, studies on the loss of mitochondrial function and/or muscle force production during adhesome dysfunction are lacking. In the present study, representative mutants displayed reduced MRAP, implying that the mitochondrial fragmentation associated with adhesome disruption represents not just structural perturbation but also functional decline. Thus, maintaining adhesomes is key to maintaining cellular energy homeostasis in *C. elegans* muscle. Similarly, as predicted, development of movement force is decreased in adhesome mutants. If attachment-mediated disruptions in cellular ATP homeostasis were sufficient to impair the ability to conduct vital cellular functions, over time an inability to maintain attachments could reduce the repair and replacement of disrupted complexes, thereby creating a vicious downward cycle in these mutants. Regardless of whether adhesion complex disruption produces weaker muscles *via* inefficient force transfer caused by disorganized sarcomeres and attachments, decreased ATP provision, or both, it is clear that the subcellular structural defects observed correlate with decreased muscle function. Although we only measured maximal mitochondrial ATP production and movement force development in 2 mutants, the identical type of subcellular structural defects are observed in our wider RNAi knockdown of the adhesome, suggesting that the integrin-adhesome as a whole is required for proper muscle cellular ATP homeostasis and generation of movement forces.

## CONCLUSIONS

Our results extend the current understanding of the breadth and complexity of integrin-adhesome maintenance of muscle structure and function *in vivo* with, strikingly, most protein components being required for normal muscle health. The dystrophic phenotypes observed in response to integrin-adhesome disruption correlate with functional impairment, with representative mutants displaying reduced mitochondrial ATP production capacity and movement force production. As such, there exists a wide range of potential molecular “Achilles heels” from which muscular dystrophy can develop (56), which will likely increase as the list of known adhesome components continues to grow (57). Further work is required to define the precise nature of muscle maintenance by integrin-adhesomes, which is characterized by a large number of protein-protein interactions, many of which are dynamic (15).

## Supplementary Material

Supplemental Data

## Abbreviations

CS: citrate synthase
GFP: green fluorescent protein
LG: linkage group
MRAP: maximal rates of mitochondrial ATP production
RNAi: RNA interference
SAGE: serial analysis of gene expression
