# Supplementary material for: The integrin-adhesome is required to maintain muscle structure, mitochondrial ATP production, and movement forces in Caenorhabditis elegans
Source: FASEB J. 2014 Dec 9;29(4):1235–46. doi: 10.1096/fj.14-259119 (PMC4396603; doi:10.1096/fj.14-259119)
Supplement: Supplemental Data [file supp_29_4_1235__index.html]

The integrin-adhesome is required to maintain muscle structure, mitochondrial ATP production, and movement forces in Caenorhabditis elegans — The integrin-adhesome is required to maintain muscle structure, mitochondrial ATP production, and movement forces in Caenorhabditis elegans — Supplemental Data 

# The integrin-adhesome is required to maintain muscle structure, mitochondrial ATP production, and movement forces in *Caenorhabditis elegans*

## Supplemental Data

**Files in this Data Supplement:**

- Supplemental Data
- Supplemental Data
- Supplemental Data
