# Supplementary figures and images for: The integrin-adhesome is required to maintain muscle structure, mitochondrial ATP production, and movement forces in Caenorhabditis elegans
Source: FASEB J. 2014 Dec 9;29(4):1235–46. doi: 10.1096/fj.14-259119 (PMC4396603; doi:10.1096/fj.14-259119)

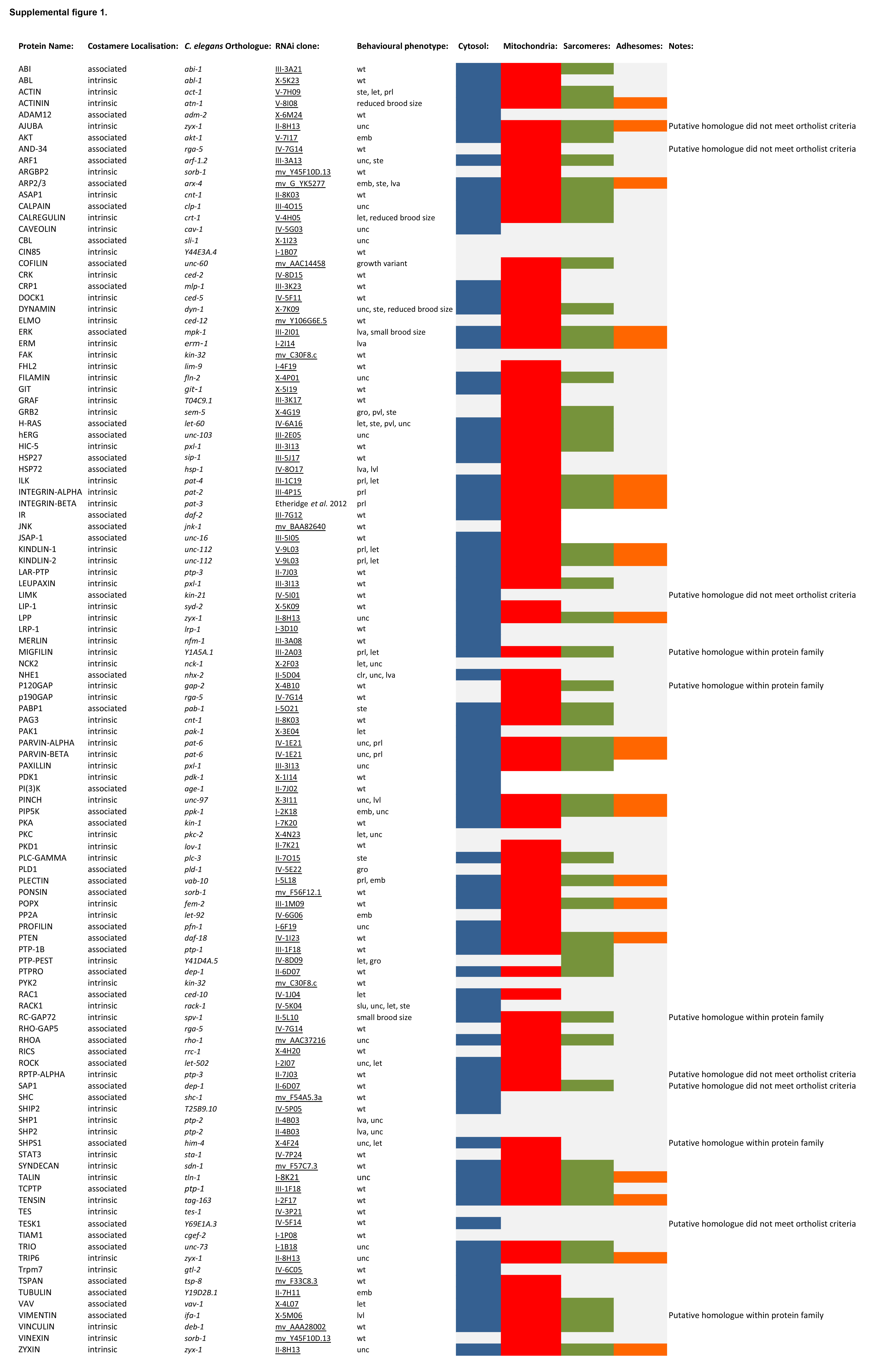

Supplement: Supplemental Data [file supp_fj.14-259119_Supplemental_Figure1.tif]

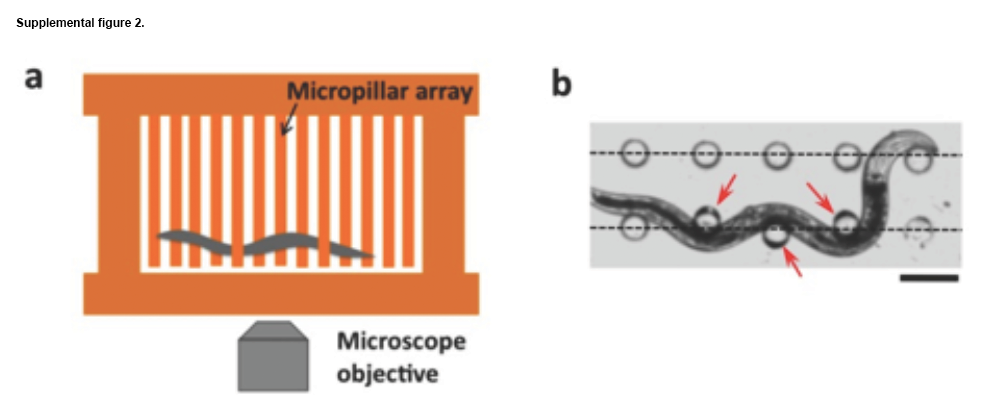

Supplement: Supplemental Data [file supp_fj.14-259119_Supplemental_Figure2.tif]
