# Supplementary material for: The integrin-adhesome is required to maintain muscle structure, mitochondrial ATP production, and movement forces in Caenorhabditis elegans
Source: FASEB J. 2014 Dec 9;29(4):1235–46. doi: 10.1096/fj.14-259119 (PMC4396603; doi:10.1096/fj.14-259119)
Supplement: Supplemental Data [file supp_fj.14-259119_Supplemental_Figures.docx]

**Suppl. Fig. 1.** Summary of RNAi-induced phenotypes. Behavioural and sub-muscular phenotypes observed for all RNAi knockdowns. Muscle phenotypes are colour coded as follows: blue, loss of cytosolic protein; red, fragmented mitochondrial network; green, disorganized sarcomeres; orange, disorganized adhesome; grey, normal. Included are *C. elegans* orthologue gene (or sequence) name for all mammalian adhesome components, adhesome component classification (intrinsic for components that permanently reside within adhesomes, associated for components that transiently locate at adhesomes), RNAi bacterial feeding vector library location, and notes on genes that did not match an Ortholist search.

**Suppl. Fig. 2.** Voluntary movement force measurement in *C. elegans*. (**a**) The micropillar device to measure the gross movement force production in *C. elegans*. (**b**) The worm crawling through the shallow chamber interacts with the pillars and deflects them. The dashed lines help in easy visualization of the deflection of the pillars. The arrows indicate the displaced pillars. Scale bar is 100 μm.
